# Cell-Assisted Lipotransfer (CAL) and Stromal Vascular Fraction (SVF) in Combination with Autologous Breast Reconstruction: A Preliminary Feasibility Case Series

**DOI:** 10.1007/s00266-026-06092-8

**Published:** 2026-07-08

**Authors:** Tung Dinh Nguyen, Hung Kim Tran, Phung Van Truong, Thao Phuong Ngoc Tran, Hoang Que Tran, Bao Cong Duong Vo, Nhu Ngoc Quynh Dinh, Nhien Do An Nguyen, Aiden Pham

**Affiliations:** 1 EMCAS Plastic Surgery Hospital, 14/27 Hoang Du Khuong Street, Hoa Hung Ward, Ho Chi Minh City, 700000 Vietnam; 2 Arizona State University, Tempe, AZ USA

**Keywords:** Cell-assisted lipotransfer, Stromal vascular fraction, Breast reconstruction, TRAM flap, LD flap, Implant reconstruction, Autologous fat grafting, MRI volumetry

## Abstract

**Background:**

Cell-assisted lipotransfer (CAL) with stromal vascular fraction (SVF) enrichment has gained increasing interest as a regenerative adjunct in breast reconstruction. However, standardized reporting of volumetric outcomes and reproducibility data remains limited. This study reports our preliminary feasibility experience integrating CAL/SVF into autologous and hybrid breast reconstruction with MRI-based volumetric assessment.

**Methods:**

Six patients undergoing breast reconstruction with CAL/SVF enrichment were prospectively evaluated. Reconstructive modalities included TRAM flap, latissimus dorsi flap, implant-based, and hybrid techniques. Intraoperative SVF isolation was performed using enzymatic digestion (0.075% type I collagenase, 30–40 minutes at 37 °C) followed by centrifugation and admixture with purified fat. Fat graft survival was assessed at 12 months using 3.0-Tesla MRI volumetry with VOI freehand segmentation. Interobserver reliability was evaluated using intraclass correlation coefficient (ICC).

**Results:**

Twelve-month fat retention ranged from 76.4% to 84.0%. No major complications occurred; one minor fat necrosis resolved without intervention. Descriptively lower retention values were observed in previously irradiated breasts. Interobserver agreement for MRI volumetry was excellent (ICC[1, 2] = 0.995; 95% CI, 0.975–0.996), supporting reproducibility of segmentation.

**Conclusions:**

CAL/SVF integration in breast reconstruction demonstrated favorable intermediate-term volumetric retention and safety within this small feasibility cohort. MRI-based volumetric analysis showed excellent interobserver reliability. Given the limited sample size and cohort heterogeneity, findings should be interpreted as preliminary and hypothesis-generating. Larger studies with extended follow-up are warranted to validate long-term durability and the impact of prior radiation.

**Level of Evidence IV:**

This journal requires that authors assign a level of evidence to each article. For a full description of these Evidence-Based Medicine ratings, please refer to the Table of Contents or the online Instructions to Authors www.springer.com/00266.

**Supplementary Information:**

The online version contains supplementary material available at https://doi.org/10.1007/s00266-026-06092-8.

## Introduction

Breast reconstruction after oncologic or prophylactic mastectomy is a critical component of breast cancer care, restoring both aesthetic form and psychosocial well-being [1]. The 2023 International Society of Aesthetic Plastic Surgery (ISAPS) global survey documented continued growth in reconstructive breast surgery worldwide, with increasing demand for natural, durable outcomes [2]. While implant-based reconstruction remains common, it is associated with well-recognized long-term complications—capsular contracture, malposition, rupture, late seroma, and breast implant-associated anaplastic large cell lymphoma (BIA-ALCL)—especially in previously irradiated or surgically compromised tissues [3, 4].

In recent years, more patients are seeking implant-free, autologous options following explantation for complications, oncologic safety, or personal preference [5]. Autologous fat grafting, introduced into reconstructive practice by Coleman, has been widely used as either a primary method or an adjunct to flap-based reconstruction [6]. It offers soft-tissue augmentation with low donor-site morbidity and regenerative benefits for irradiated skin, but its unpredictable long-term retention**—**reported to range from 25% to 80%—remains a limitation [7–9]. Factors influencing graft survival include harvest and processing techniques, injection methods, recipient bed vascularity, and patient history of radiotherapy [10].

Cell-assisted lipotransfer (CAL)**,** first described by Matsumoto et al. in 2006 [11], enriches lipoaspirate with stromal vascular fraction (SVF)**—**a heterogeneous cell population containing adipose-derived stem/stromal cells (ASCs), endothelial progenitor cells, pericytes, and immune-regulatory cells [12, 13]. SVF is thought to improve graft survival via enhanced angiogenesis, reduced ischemia-induced apoptosis, and improved extracellular matrix integration [14]. A 2025 network meta-analysis of 31 randomized trials (n = 1,656) found that ADSC-assisted fat grafting had the highest probability of graft survival (SUCRA 82.17%), while SVF-assisted grafting achieved moderate but significant improvement over conventional lipofilling (SUCRA 65.14%) [15].

Recent clinical safety data further support CAL in reconstructive settings. The LIPO MULTI-SVF study (2025) employed a novel staggered SVF delivery strategy in 28 patients, using an average of 160.8 ± 17 mL of fat graft and 134.8 ± 16.4 mL of SVF source material. Over a median follow-up of 2.0–5.8 years, no oncologic recurrences or procedure-related infections were reported (1). In the challenging subgroup of irradiated breasts, a 2023 systematic review concluded that CAL significantly improves contour and aesthetic outcomes without increasing recurrence risk, although volume retention is typically lower than in non-irradiated cases (*p* < 0.05) [3].

## Rationale in the Southeast Asian Context

The relevance of CAL with SVF in Southeast Asia is underscored by several regional factors. First, the age of breast cancer onset in many Southeast Asian countries is approximately a decade younger than in Western populations—often affecting women in their 30s and 40s, who have longer postoperative life expectancy and higher aesthetic demands [16]. Second, the high rates of postmastectomy radiotherapy in resource-limited settings create challenging reconstructive conditions, making regenerative adjuncts particularly valuable [17]. Third, the tropical climate and higher prevalence of infection-prone environments necessitate techniques with robust tissue healing and vascularity support. Finally, economic considerations play a significant role—CAL integrated into flap-based reconstruction allows for single-stage procedures, reducing the need for multiple revision surgeries and thereby lowering the overall treatment burden [18]. These factors together make Southeast Asia a fertile ground for the adoption and study of hybrid regenerative reconstructive strategies. Prior reports indicate that fat graft retention typically stabilizes by 6–12 months postoperatively [6, 11].

### Study Objective

This study reports a preliminary six-patient feasibility case series incorporating cell-assisted lipotransfer (CAL) with stromal vascular fraction (SVF) enrichment across diverse reconstructive contexts, including TRAM flaps, latissimus dorsi flaps, implant-based reconstructions, and hybrid procedures. Each SVF preparation underwent laboratory quality control, including assessment of total nucleated cell count, viability, sterility, endotoxin levels, and mycoplasma status.

The primary objective was to evaluate procedural feasibility, cellular quality parameters, safety, and MRI-based intermediate-term volumetric outcomes following CAL/SVF integration in breast reconstruction. A secondary objective was to describe the adaptability of this approach across heterogeneous reconstructive scenarios, including previously irradiated or high-risk tissues.

## Patients and Methods

### Study Design

This retrospective case series was conducted at EMCAS Plastic Surgery Hospital (Ho Chi Minh City, Vietnam) between January 2016 and December 2024. All consecutive female patients undergoing autologous or hybrid breast reconstruction incorporating cell-assisted lipotransfer (CAL) with stromal vascular fraction (SVF) enrichment were eligible for inclusion. The study received institutional ethics approval, and all participants provided written informed consent for surgical treatment and anonymized data use [1] .

### Inclusion and Exclusion Criteria

Eligible patients included those undergoing:

- Autologous flap breast reconstruction (TRAM or LD flap) with CAL/SVF [5, 19, 20].
- Implant-based or hybrid reconstruction with CAL/SVF augmentation [18, 21]
- Secondary revision surgery after implant removal or oncologic mastectomy

Exclusion criteria were:

- Active breast cancer or local recurrence at time of reconstruction [4]
- Active infection or unhealed wound in the recipient area
- Uncontrolled systemic disease or coagulopathy
- Pregnancy or planned pregnancy within 12 months

### Preoperative Assessment

All patients underwent:

- Detailed oncologic history review (tumor stage, adjuvant therapies, recurrence status) [5]
- Clinical breast examination, donor-site assessment, and documentation of skin quality
- Preoperative imaging (mammography, ultrasound, or MRI) when indicated [6]
- Baseline laboratory studies including complete blood count and coagulation profile

## Surgical Protocol

### Autologous TRAM Flap Reconstruction

The pedicled TRAM flap was designed over the lower abdomen, incorporating skin, subcutaneous tissue, rectus abdominis muscle, and anterior rectus sheath [19]. Dissection preserved the superior epigastric vessels and musculocutaneous perforators [7]. The flap was tunneled to the mastectomy site, de-epithelialized as required, and shaped for optimal breast mound contour [9]. Donor-site closure included polypropylene mesh reinforcement to prevent hernia or bulging [19].

### Latissimus Dorsi (LD) Flap Reconstruction

The pedicled LD flap, based on the thoracodorsal vessels, was harvested with or without a skin paddle, tunneled to the anterior chest, and inset into the mastectomy pocket [20].

### Implant-Based Reconstruction

**Fig. 1:**
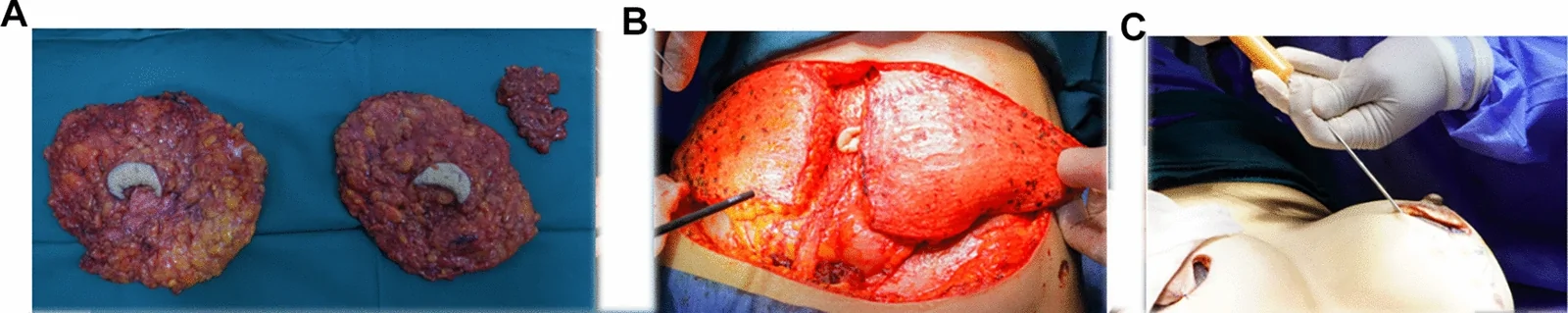
**A**: Bilateral mastectomy,** B**: TRAM flap,** C**: Fat grafting after breast implant reconstruction

For primary reconstruction, a prepectoral or dual-plane pocket was created following mastectomy [21]. In revision cases, total or partial capsulectomy was performed to remove fibrotic tissue [14]. Silicone gel implants were selected based on intraoperative sizer evaluation for symmetry (Fig. 1) [21].

### CAL/SVF Preparation and Fat Grafting

**Fig. 2:**
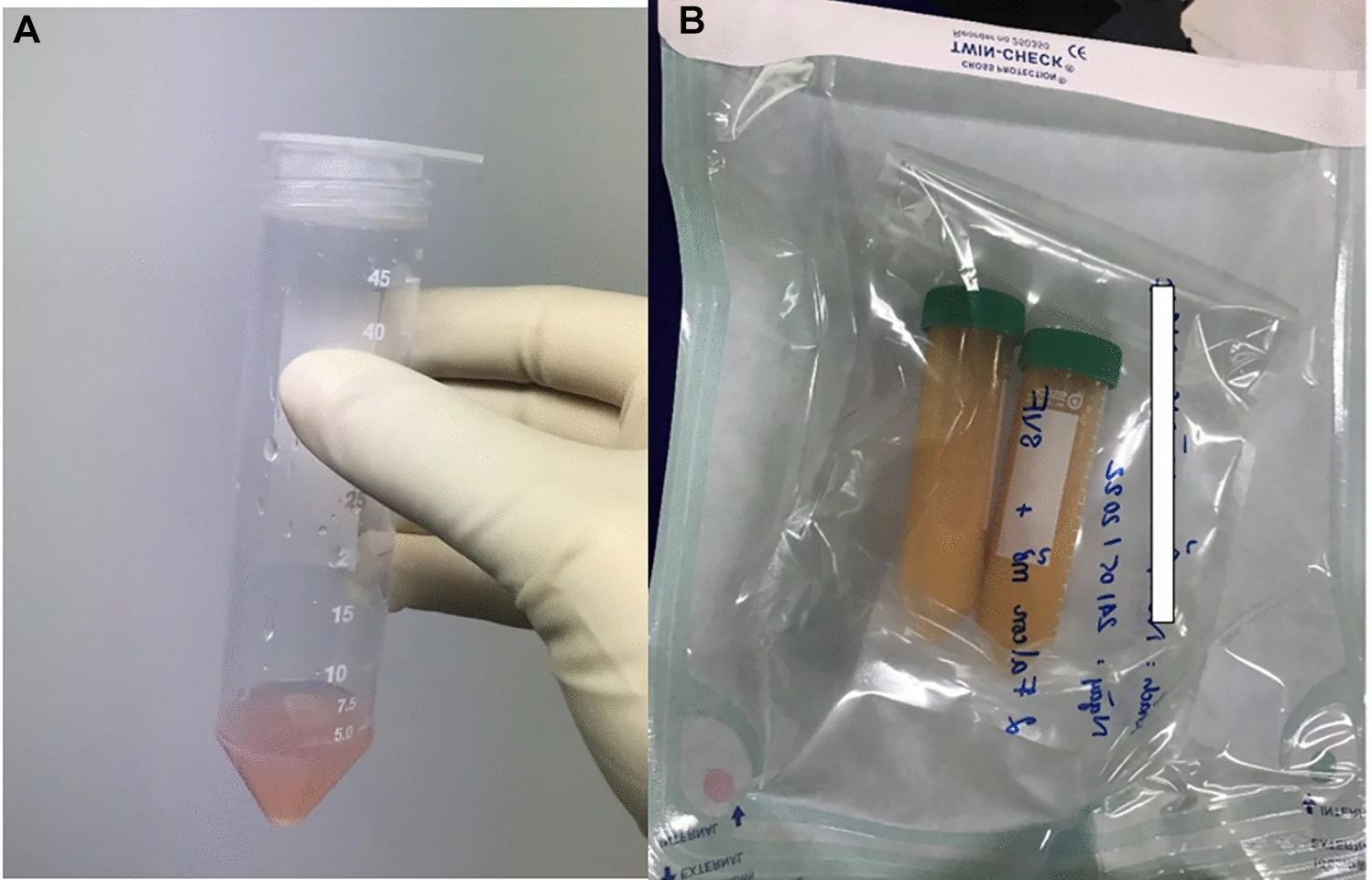
Intraoperative isolation of stromal vascular fraction (SVF) and preparation of CAL-enriched fat graft. **A** SVF pellet following collagenase digestion and centrifugation of lipoaspirate. **B** Purified fat mixed with isolated SVF in sterile preparation prior to multi-plane injection for breast reconstruction

Autologous lipoaspirate was obtained using 3- to 4-mm blunt cannulas under low pressure from the abdomen, flanks, or thighs [11]. SVF was isolated via enzymatic digestion (collagenase type I), followed by centrifugation of 0.075% (w/v) for 30–40 minutes at 37°C under sterile conditions with gentle agitation [10, 14]. Following enzymatic neutralization, the digest was centrifuged at 1200 g for 5 minutes. The stromal vascular fraction pellet was subsequently isolated, washed with sterile saline, and resuspended in lactated Ringer’s solution prior to admixture with purified fat for reinjection [15]. CAL-enriched fat was injected in micro-aliquots (approximately 0.1–0.2 mL per pass) using a multi-plane tunneling technique involving subcutaneous and intramuscular planes to optimize surface area contact and reduce central necrosis risk (Fig. 2) [9, 13] .

A schematic illustration showing:

Harvest → Digestion → Centrifugation → Cell Count → Mixing → Injection

### Postoperative Care

Closed suction drains were placed at donor and recipient sites, removed when drainage was <30 mL/24 h [19]. Compression garments were worn for 4–6 weeks at donor sites. Prophylactic antibiotics were administered perioperatively. Clinical follow-up was scheduled at 1, 3, and 6 months postoperatively.

### Fat Graft Survival Assessment

MRI volumetric assessment was performed at 12 months postoperatively using a 3.0-Tesla system equipped with a dedicated bilateral breast coil. Axial T1-weighted sequences with 1–3 mm slice thickness were obtained to allow high-resolution volumetric segmentation. No intravenous contrast was administered for volume calculation.

Breast volume segmentation was conducted using the VOI freehand tool on the MRI workstation, consistent with previously described MRI-based volumetric techniques for fat graft assessment [22]. Anatomical boundaries were predefined as follows: superiorly at the clavicle, inferiorly at the inframammary fold, medially at the sternal border, and laterally at the anterior axillary line. Chest wall musculature and non-grafted tissue were excluded from the segmented region. Total volume was calculated by summation of segmented slice areas multiplied by slice thickness [22].

Two independent observers performed volumetric analysis in a blinded fashion. Interobserver reliability was assessed using the intraclass correlation coefficient (ICC) based on a two-way random-effects model with absolute agreement for single measurements (ICC[1, 2]). Because two patients underwent bilateral reconstruction, each breast was treated as an independent unit for reliability analysis (n = 8 breasts).

**Fig. 3:**
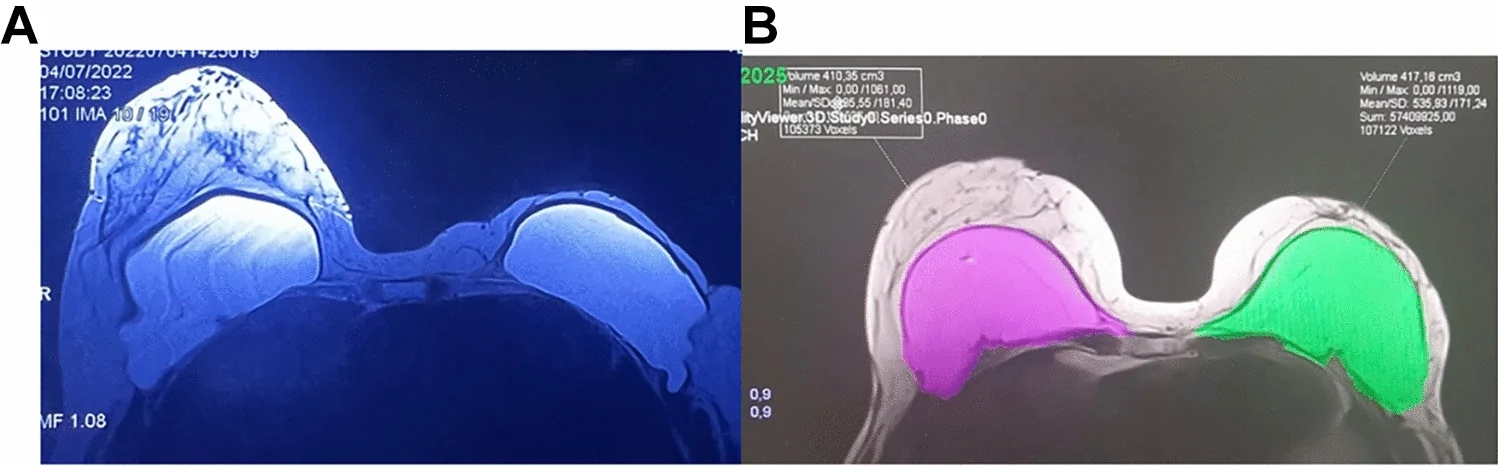
**A**: MRI before surgery,** B**: Assessement V2 volume by MRI in 12 months after surgery

Fat retention (%) was calculated using the formula:

$${\text{Retention }}\left( \% \right) = \left( {{\mathrm{V2}}/{\mathrm{V1}}} \right) \times {1}00$$

where V1 represents intraoperative graft volume and V2 represents MRI-measured volume at 12 months (Fig. 3) [22].

## Results

### Patient Characteristics

Six female patients aged 33–52 years (mean age 40.3 years) underwent breast reconstruction incorporating cell-assisted lipotransfer (CAL) with stromal vascular fraction (SVF) enrichment between 2016 and 2024 (Table 1). Four patients had a history of adjuvant radiotherapy, and one patient was BRCA2-positive and underwent contralateral prophylactic mastectomy [4, 16].

**Table 1.** Patient characteristics, SVF parameters, and 12-month volumetric outcomes

Case	Age (y)	Radiation	Reconstruction Modality	SVF cells (×10⁶)	Viability (%)	Fat Graft Volume (mL)	MRI Volume V2 (mL)	Retention (%)	Complications	Follow-up (mo)
1	33	No	LD revision + contralateral implant	1.9	97.8	100	82	82.0	None	12
2	38	Yes	TRAM (right) + implant (left)	5.1	98.0	100	76	76.4	None	12
3	52	Yes	Bilateral TRAM after implant removal	2.0	97.3	150 / 100	121 / 81	80.7	Minor fat necrosis (resolved)	12
4	36	Yes	Bilateral TRAM + implant	2.3	98.2	100	78	78.0	None	12
5	35	No	Implant-based reconstruction	2.0	98.8	150 / 100	126 / 84	84.0	None	12
6	48	Yes	Bilateral TRAM	2.6	97.6	100	79	79.0	None	12

MRI-based volumetric assessment was performed at 12 months postoperatively. Retention (%) was calculated as V2/V1 ×100. All SVF preparations met predefined sterility and endotoxin safety specifications. One patient (Case 3) developed a small area of fat necrosis that resolved without intervention.

Interobserver agreement for MRI volumetry was excellent (ICC[1, 2] = 0.995; 95% CI, 0.975–0.996), supporting the reproducibility of VOI-based segmentation in this cohort..

### SVF Quality Control

All SVF preparations met the predefined laboratory safety and viability standards [10, 14]:

- *Cell viability*: >97% in all cases (specification >90%)
- *Sterility*: All cultures negative
- *Bacterial endotoxin*: <0.25 EU/mL in all cases
- *Mycoplasma*: Negative in all cases

### Early Clinical Outcomes

**Fig. 4:**
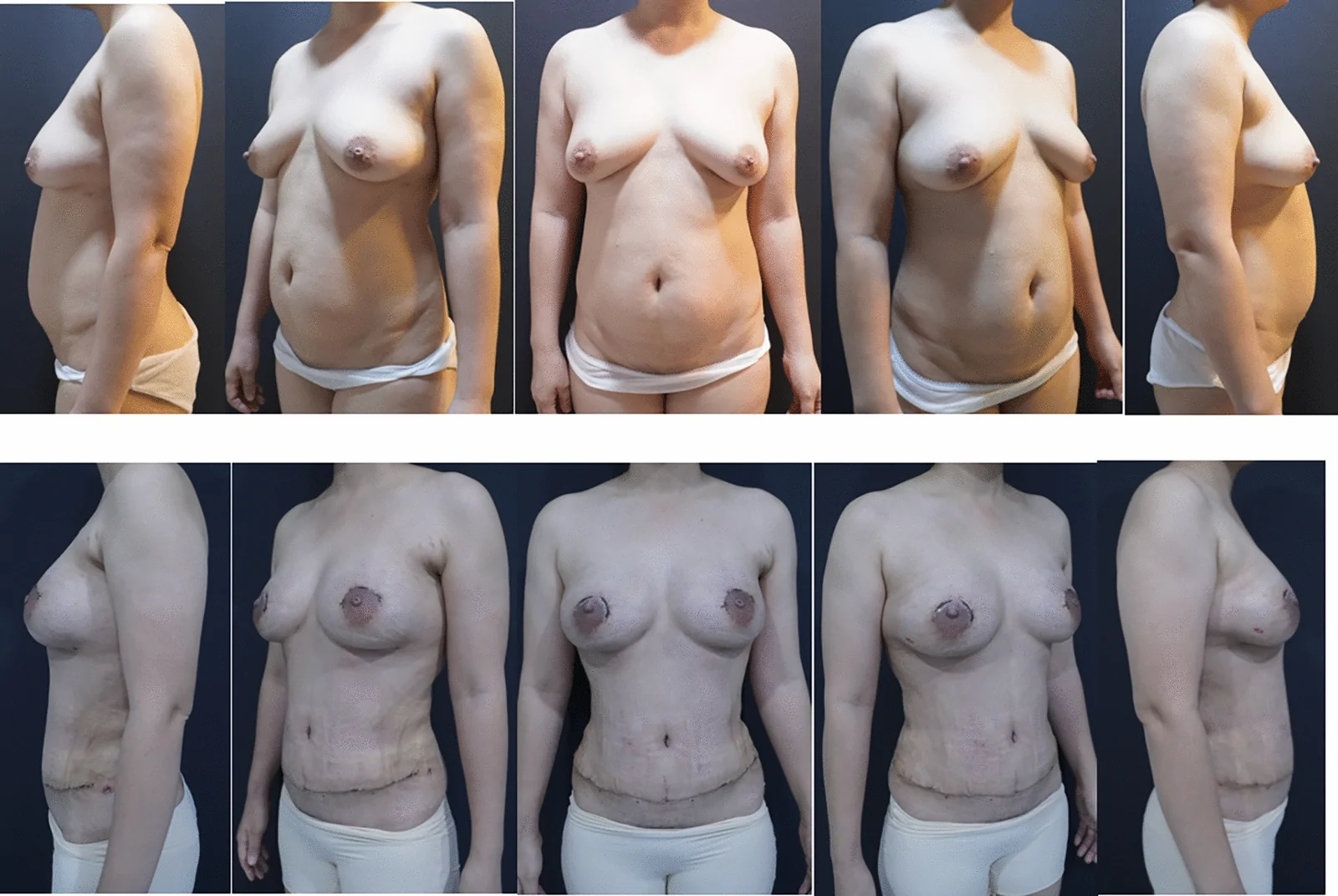
Before and after 3 weeks postsurgery ( case 6)

**Fig. 5:**
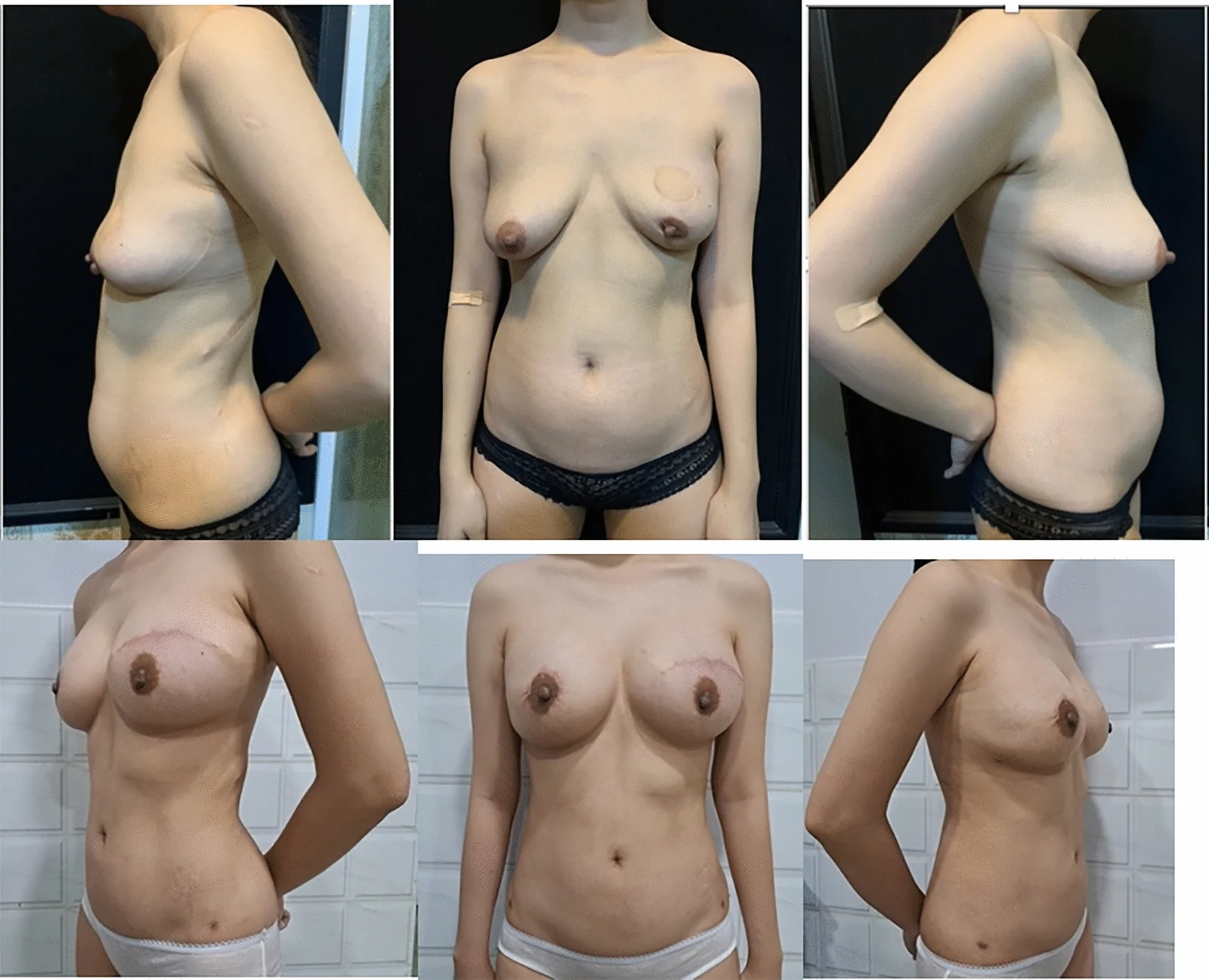
Before and 12 months postsurgery (case 1)

**Fig. 6:**
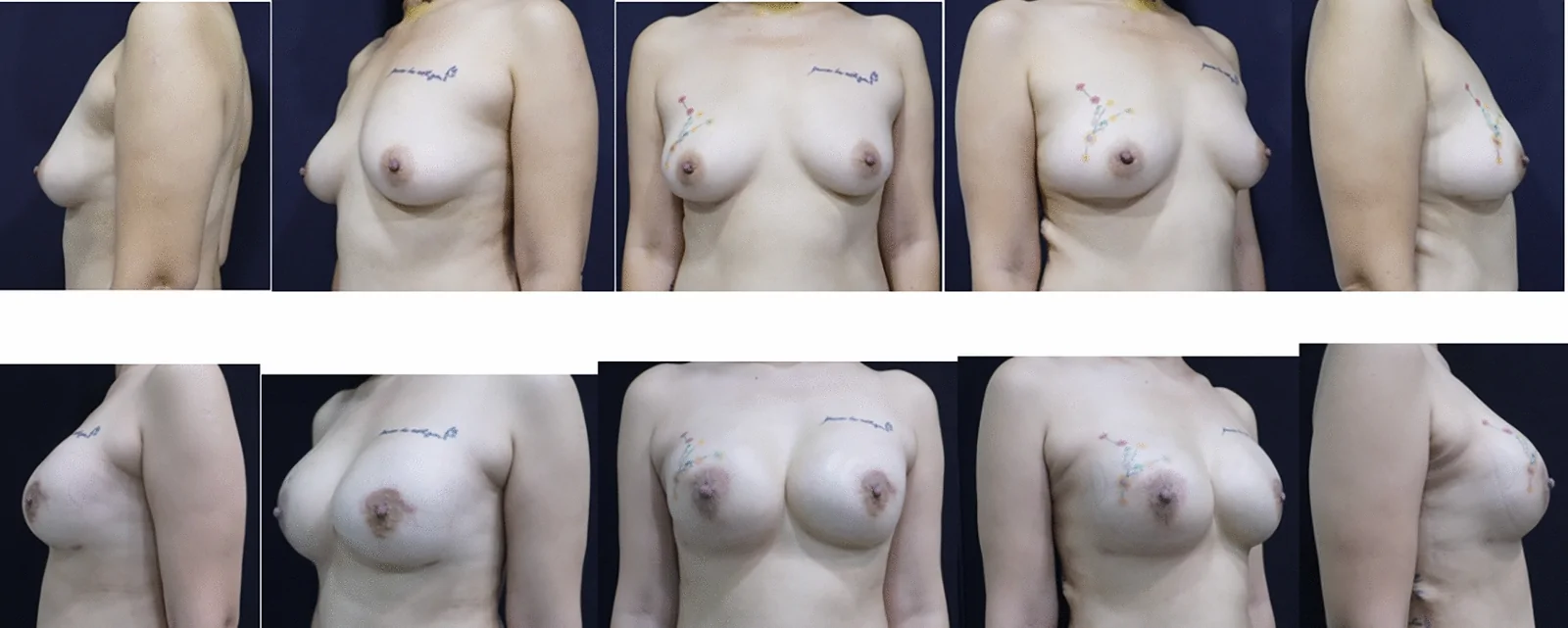
Before and 6 months postsurgery (case 5)

- *Symmetry and Contour:* All patients achieved satisfactory symmetry at 6 months, with improved upper pole fullness and natural medial cleavage in hybrid reconstructions [5, 18].
- *Complications:* No flap losses, implant exposures, infections, or seromas occurred, consistent with complication rates reported in large CAL/SVF series [1, 3, 12]. Minor fat necrosis occurred in one patient (case 3), presenting as a small palpable nodule that resolved without intervention [11, 13] .
- *Volume Stability:* At 12-month MRI follow-up, calculated fat graft retention ranged from 76.4% to 84.0% (mean 80.1%), similar to retention rates reported in recent prospective trials of CAL (Figs. 4, 5 and 6) [1, 17, 22] . Higher retention was observed in non-irradiated breasts [3, 4] and in flap-based reconstructions compared to implant-based cases [5, 18]
- *Donor Site:* All abdominal donor sites healed without hernia or bulging, aided by mesh reinforcement in TRAM cases [19] .

## Discussion

This case series demonstrates the feasibility, safety, and efficacy of cell-assisted lipotransfer (CAL) with stromal vascular fraction (SVF) enrichment in complex breast reconstruction scenarios in a Southeast Asian setting. Our six-patient cohort achieved a mean 12-month fat graft retention of 80.1%**,** with no major complications and minimal donor-site morbidity. These outcomes align closely with the LIPO MULTI-SVF Study** (**Agostini et al., 2025), which reported mean retention rates of 78–82% at one year in oncologic reconstruction [1].

The heterogeneity of reconstructive techniques (TRAM flap, LD flap, implant-based, and hybrid approaches) reflects the variability of clinical indications encountered in reconstructive breast surgery. However, within the context of a six-patient case series, this diversity precludes meaningful subgroup comparison and limits generalizability. The purpose of including multiple reconstructive modalities was to demonstrate the adaptability of CAL/SVF integration rather than to compare reconstructive outcomes between techniques.

### Retention Rates Compared to Literature

In our series, fat graft survival was descriptively higher in non-irradiated breasts and in flap-based reconstructions than in irradiated or purely implant-based cases. Observed retention rates in this limited cohort were consistent with previously published CAL-assisted lipotransfer data [9, 13].

This mirrors findings from Wu et al. (2023), where irradiation was associated with reduced graft retention despite regenerative adjuncts [3]. TRAM flap reconstructions, which provide a well-vascularized recipient bed, may enhance adipocyte integration and survival compared to silicone implant pockets [5, 18].

Our retention rate also compares favorably with conventional fat grafting without SVF, which has been reported to yield approximately 50–70% volume retention at one year [11, 13]. The improved outcome may be attributable to the angiogenic and anti-inflammatory properties of SVF-derived adipose stromal cells, which promote neovascularization and mitigate hypoxic adipocyte loss [9, 10, 15].

The potential impact of radiation on adipose graft survival has been described in prior studies, which attribute reduced retention to stromal fibrosis, vascular compromise, and impaired adipogenesis [3, 4].

In this cohort, descriptively lower retention rates were observed in previously irradiated breasts. However, given the limited sample size and heterogeneity of reconstructive modalities, this observation should be interpreted cautiously. While prior literature suggests that radiation-induced fibrosis and microvascular compromise may impair adipocyte survival [3, 4], the present study was not powered to determine the independent effect of radiation on graft retention. These findings should therefore be considered hypothesis-generating and warrant validation in larger, controlled studies.

MRI-based volumetric assessment was performed at 12 months, as prior studies suggest that the majority of adipocyte resorption occurs within the first 3–6 months following grafting, with relative stabilization thereafter [6, 11]. While 12-month evaluation provides meaningful intermediate-term data, it does not fully establish long-term durability. Continued longitudinal follow-up of this cohort is ongoing to assess volumetric stability beyond the first postoperative year.

### Oncologic Safety

No cases of local recurrence were observed during the follow-up period, consistent with published evidence that autologous fat grafting and CAL do not increase breast cancer recurrence risk [4, 16]. Our patient cohort included one BRCA2-positive case undergoing prophylactic contralateral mastectomy, reinforcing CAL’s applicability in high-risk populations when performed after complete oncologic clearance [16].

### Clinical Relevance in Southeast Asia

Breast reconstruction uptake in Southeast Asia remains lower than in Western countries, partly due to cultural perceptions, cost barriers, and limited access to microsurgical expertise [2]. Hybrid techniques such as CAL with SVF can offer:

1. Enhanced aesthetic outcomes without requiring advanced microsurgical skills for every case, making it adaptable for centers without full microsurgical capability.
2. Improved results in irradiated tissues**,** which is relevant since postmastectomy radiotherapy rates in Asian breast cancer patients can be as high as 50% [3, 4].
3. Smaller donor-site burden, a consideration in leaner Asian body types where large-volume flaps may not be possible [5].

### Technical Considerations

Our protocol followed a low-pressure harvest technique, immediate SVF isolation under sterile conditions, and micro-aliquot injection to minimize necrosis [10, 11, 14]. Polypropylene mesh reinforcement was used in all TRAM donor sites, preventing abdominal bulging or hernia [19].

MRI volumetry at 12 months provided an objective measure of retention, in line with recommendations from Ozturk et al. (2023) [22]. This approach avoids overestimation of volume survival often seen with photographic or anthropometric assessment.

### Limitations

The small sample size limits statistical inference, and our follow-up period of 12 months may not capture long-term volume stability or oncologic outcomes. Additionally, while SVF cell viability exceeded 97% in all cases, we did not perform phenotypic characterization of cell subpopulations, which may influence graft behavior [10, 14]. The principal limitation of this study is the small sample size (n = 6), which precludes statistical inference and limits generalizability. As a Level IV case series, the findings should be interpreted as preliminary observations focused on feasibility and safety rather than definitive efficacy conclusions. The absence of a matched non-CAL control cohort limits the ability to determine incremental benefit attributable to SVF enrichment. Future prospective studies incorporating randomized or matched comparative designs will be essential to clarify whether CAL provides statistically significant improvement over conventional fat grafting techniques.

Although interobserver agreement for MRI volumetry was excellent, the reliability analysis was derived from a limited number of reconstructed breasts (n = 8), consistent with the feasibility design of this study. Larger cohorts would further strengthen validation of volumetric reproducibility.

The limited number of irradiated versus non-irradiated breasts precludes meaningful subgroup analysis regarding the independent effect of radiation therapy.

The current follow-up duration of 12 months limits assessment of long-term graft durability and delayed volume changes. Extended follow-up in larger cohorts will be necessary to confirm sustained volumetric stability.

## Conclusion

In this preliminary six-patient feasibility case series, CAL with SVF enrichment demonstrated procedural feasibility, high cellular viability, sterility compliance, and favorable intermediate-term volumetric retention as assessed by standardized MRI analysis. These findings should be interpreted as descriptive and hypothesis-generating within the context of a small, heterogeneous cohort. Larger controlled studies with homogeneous populations and extended follow-up are required to determine long-term durability and comparative efficacy.

## Supplementary Information

Below is the link to the electronic supplementary material.

Supplementary file1 (MP4 100970 kb)
